# Targeting of conserved spike epitopes enables broad antibody neutralisation of diverse bat sarbecoviruses

**DOI:** 10.1371/journal.pbio.3003882

**Published:** 2026-10-01

**Authors:** Ayush Upadhyay, Jeffrey Seow, Yilmaz Alguel, Joseph Newman, Nazia Thakur, Abigail L. Hay, Jerry C. H. Tam, Andrea Nans, Richard J. Orton, Dalan Bailey, Peter Cherepanov, Katie J. Doores

**Affiliations:** 1 Department of Infectious Diseases, School of Immunology & Microbial Sciences, King’s College London, London, United Kingdom; 2 Chromatin Structure and Mobile DNA Laboratory, The Francis Crick Institute, London, United Kingdom; 3 The Pirbright Institute, Woking, Surrey, United Kingdom; 4 Structural Biology Science Technology Platform, The Francis Crick Institute, London, United Kingdom; 5 MRC-University of Glasgow Centre for Virus Research, The University of Glasgow, Glasgow, United Kingdom; 6 Department of Infectious Disease, South Kensington Campus, Imperial College London, London, United Kingdom; Washington University School of Medicine, UNITED STATES OF AMERICA

## Abstract

The continuing evolution of SARS-CoV-2 variants of concern, and the increasing spillover potential of sarbecoviruses into the human population presents an important and urgent need to discover cross-reactive monoclonal antibodies (mAbs) for future therapeutic use and identify conserved neutralising epitopes that can be used for rationale design of broadly protective sarbecovirus vaccines. Here we study the neutralising epitopes on WIV-1 Spike of three mAbs that confer broad sarbecovirus and SARS-CoV-2 variant neutralisation, including XEC, JN.1, and XFG. mAb V1WT_06 binds a highly conserved RBD site V epitope that is mediated by the heavy chain alone. V1WT_06 contact residues are highly conserved in circulating viruses suggesting that the epitope is evolutionarily and functionally constrained. mAbs V1WT_41 and VA14_26 bind overlapping RBD class 4 epitopes with differing angles of approach that impact on the degree of ACE2 competition. We show that neutralisation by these mAbs is maintained when virus entry is via Japanese horseshoe bat and Halcyon horseshoe bat ACE2s. These mAbs are ideal candidates for therapeutic antibody development and inform the rational design of pan-betacoronavirus vaccines.

## Abstract 2

The increasing spillover risk of sarbecoviruses into humans stresses the urgent need to identify cross-reactive antibodies with therapeutic potential. This study uncovers the mechanisms of broad sarbecovirus cross-reactivity of three human antibodies that can inform the rational design of therapeutic antibodies and pan-coronavirus vaccines.

## Introduction

The virally encoded sarbecovirus surface glycoprotein, Spike, is critical for host cell attachment and entry, and is the major target for neutralising antibodies (nAbs) [1]. Spike is the predominant antigen target for both immunogen development and for antibody-based therapeutics against COVID-19. An increasing spillover potential of sarbecoviruses, and the continuing prevalence of emerging SARS-CoV-2 variants, presents an important and urgent need for identifying both cross-reactive monoclonal antibodies (mAbs) for therapeutic use and conserved neutralising epitopes that can inform rational design of broadly protective sarbecovirus vaccines. Since the beginning of the COVID-19 pandemic, the population has generated a high degree of humoral and cellular immunity to SARS-CoV-2, arising in response to both infection and vaccination [2–4]. An understanding of potential population-wide protection against new and emerging sarbecoviruses from animal hosts will be critical for future pandemic preparedness.

Sarbecoviruses can differ in their receptor usage for host cell entry. Whilst some viruses target a host-specific receptor for entry, others can utilise a range of receptors resulting in a wider host diversity [5–7]. Sarbecoviruses that use human angiotensin-converting enzyme 2 (ACE2) for entry are of most concern for zoonosis events as would likely require minimal adaptation. However, rapid virus adaptation [5] and the potential for expanded host range means that nAbs that also target animal viruses with zoonotic potential are of relevance and importance for future pandemic preparedness [5]. Furthermore, the continued selective pressure of humoral immunity is demonstrated by the on-going evolution of SARS-CoV-2 variants and the appearance of additional Spike mutations that reduce the potency of immune sera and escape therapeutic mAbs [8–12]. Therefore, there remains an urgent need to identify SARS-CoV-2 mAbs that can resist viral escape for development of improved and future-proofed antibody-based SARS-CoV-2 therapies.

The receptor binding domain (RBD) of SARS-CoV-2 Spike interacts with the ACE2 receptor on targets cells and is one of the dominant targets of nAbs generated in response to infection and vaccination [13–15]. Several epitopes on RBD have been identified. A dominant epitope is the highly variable receptor binding motif (RBM), where antibodies function by directly inhibiting the interaction of Spike with the ACE2 receptor (referred to as Class 1 and Class 2 [14]). SARS-CoV-2 mutations frequently arise in this epitope due to the selective pressure of neutralising antibodies driving immune evasion [10,16]. Other RBD epitopes distal to RBM include the CR3022 cryptic epitope (referred to as class 4 [14]), the N343-site (referred to as Class 3 [14]) and RBD site V [17].

We have shown that sarbecoviruses with a broad ACE2 tropism that includes human ACE2 (hu-ACE2) can be neutralised by SARS-CoV-2 convalescent and vaccinee sera [18–20]. In this study, we also observed that some bat sarbecoviruses with more specialist ACE2 tropism were also weakly neutralised by these sera. Through screening a panel of neutralising mAbs isolated from donors who were convalescent, vaccinated, or experienced a breakthrough infection (BTI) [8,21,22], we identified several RBD-specific mAbs with broad binding and neutralising activity against bat sarbecoviruses belonging to multiple clades, including clades 1a, 1b, 3 and 5.

Here, we structurally characterised the RBD epitopes of three mAbs with the broadest SARS-CoV-2 variant neutralisation and sarbecovirus binding and studied their mechanisms of neutralisation in the context of ACE2 receptors from different hosts. We show that these mAbs target the RBD site V or class 4 epitopes and achieve their neutralisation breadth through binding to regions that are highly conserved between SARS-CoV-2 variants and bat sarbecoviruses. We identify a unique potently neutralising antibody that approaches the RBD site V with a binding mechanism and angle of approach that could potentially facilitate greater accessibility to the cryptic epitope. The binding epitopes of these mAbs make them ideal candidates for therapeutic antibody development and inform the rational design of pan-betacoronavirus vaccines.

## Results

### Cross-reactive mAbs bind noncompeting epitopes on SARS-CoV-2 Spike

We identified three RBD-specific mAbs isolated from either a AZD1222 vaccinated individual (VA14_26) [22] or an individual, who was infected with the delta variant after BNT162b2 vaccination (V1WT_06 and V1WT_41) [8]. The three mAbs displayed broad cross-binding activity against RBDs of SARS-CoV-2 variants and related bat sarbecoviruses including hu-ACE2 using viruses (BANAL-20-52, BANAL-20-236, WIV-1 and Rs4231), as well bat specific viruses that have more restricted bat ACE2 usage (Rc-o319, and RhGB07) by surface plasma resonance (SPR) (S1A Fig) [20,23,24] and Spikes (WIV-1, Rs4231, RpYN06, Rf1/2004, RP3/2004, and RacCS203) by flow cytometry (S1B, S1C Fig). Fab versions of the three mAbs were cloned and expressed to facilitate detailed functional and structural characterisation of their neutralising epitopes. When comparing the binding of the Fab and IgGs against the RBDs of sarbecoviruses by ELISA, there was a reduction in Fab Spike binding (Fig 1A) for V1WT_41, VA14_26 and V1WT_06 for some RBDs indicating that multivalency is important for binding to specific sarbecoviruses.

**Fig 1:**
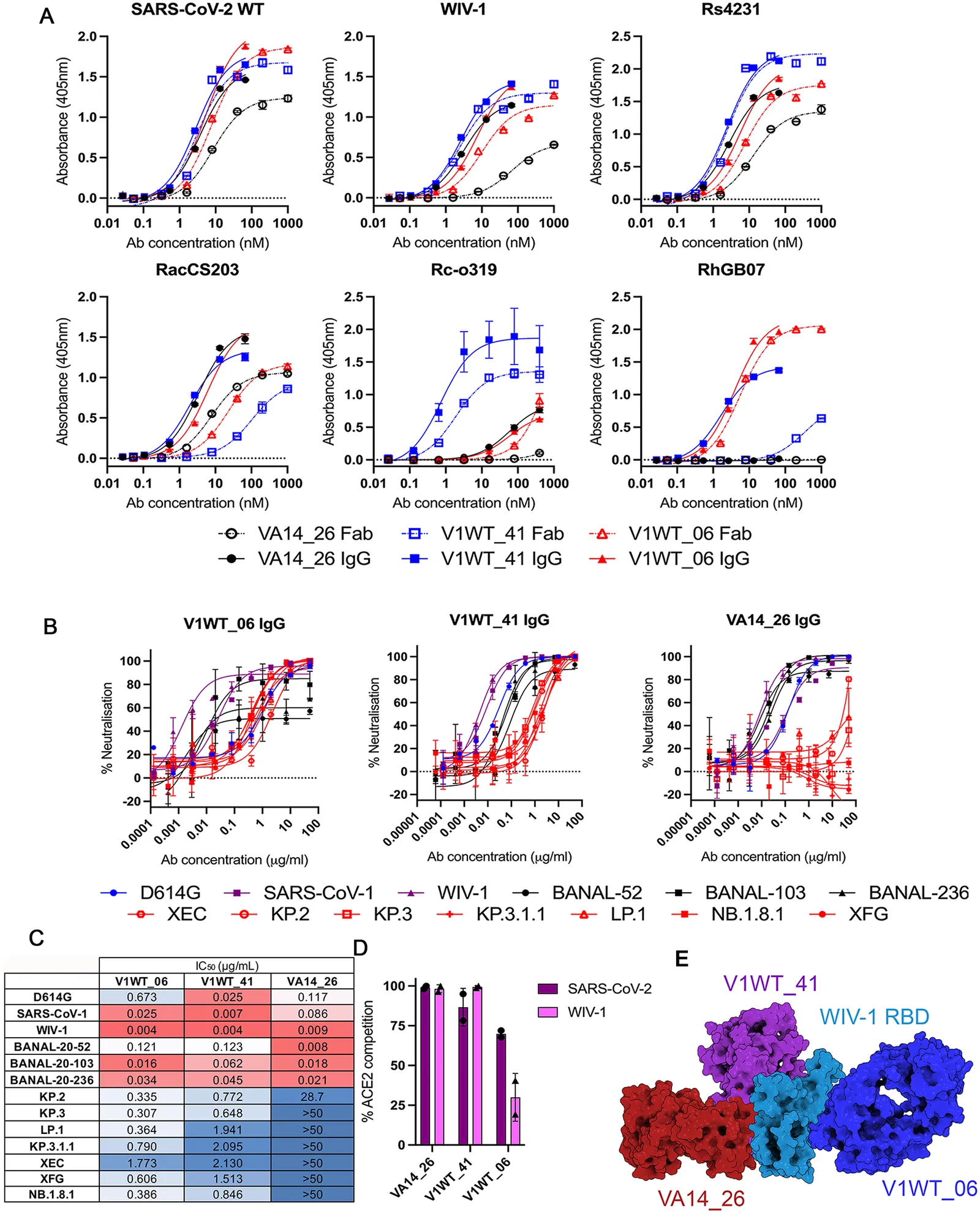
Binding and functional activity of V1WT_06, V1WT_41 and VA14_26. **A)** Binding of IgG and Fab to recombinant RBDs of Sarbecoviruses from different clades by ELISA including SARS-CoV-2 (clade 1b), WIV-1 (clade 1a), Rs4321 (clade 1a), RacCS203 (clade 2), Rc-o319 (clade 5), and RhGB07 (clade 3). Experiments were run in duplicate and repeated twice. A representative assay is shown. Fab binding is shown with the dotted line and IgG binding with the full line. **B)** Neutralising activity of IgGs against SARS-CoV-2 and variants of concern as well as SARS-CoV-1 and WIV-1. **C)** IC_50_ values for neutralisation against SARS-CoV-2 variants of concern and other sarbecoviruses that utilise human-ACE2. For A–C, the mean value is plotted with error bars showing ± standard deviation (s.d.). **D)** Ability of IgGs to inhibit the interaction between cell surface human ACE2 and soluble SARS-CoV-2 and WIV-1 RBDs. IgGs were pre-incubated with fluorescently labelled RBD before addition to HeLa-ACE2 cells. The percentage reduction in mean fluorescence intensity is reported. Experiments were performed in duplicate. **E)** Superimposed 3D structures of the V1WT_06 (dark blue), V1WT_41 (purple), and VA14_26 (red) around WIV-1 RBD (light blue, from PDB 8WLZ). The data underlying this Figure can be found in S1 Data.

Neutralisation against recent SARS-CoV-2 variants was tested including KP.2, KP.3, LP.1, KP.3.1.1, NB.1.8.1, XEC, and XFG and compared to the cross-neutralisation of human-ACE2 using SARS-CoV-1, WIV-1, BANAL-20-52, BANAL-20-103, and BANAL-20-236 (Fig 1B, 1C). V1WT_06 retained both breadth and potency against all VOCs and sarbecoviruses tested. Whilst V1WT_41 retained breadth, there was between a 25- and 85-fold reduction in potency against the seven SARS-CoV-2 variants tested. VA14_26 was most impacted by Spike mutations and was only able to weakly neutralise KP.3. Interestingly, the three mAbs displayed higher neutralisation potency against the WIV-1 pseudotype compared to D614G and other SARS-CoV-2 variants.

To gain insight into the mechanisms of mAb cross-neutralisation, we performed an ACE2 competition assay using HeLa cells stably expressing hu-ACE2 and recombinant SARS-CoV-2 or WIV-1 RBDs (Fig 1D). V1WT_41 and VA14_26 competed with hu-ACE2 for binding to both SARS-CoV-2 and WIV-1 RBDs. In contrast, V1WT_06 showed only a partial hu-ACE2 competition with SARS-CoV-2 RBD and minimal competition with WIV-1 RBD (Fig 1D).

### Structural definition of mAb epitopes on WIV-1 Spike

To gain a detailed understanding of how the mAbs derive their broad cross-recognition, we imaged pre-fusion stabilised WIV-1 Spike ectodomain in the presence of the corresponding Fab fragments by cryo-EM. The WIV-1 Spike was stabilised in a pre-fusion state by K969P and V970P mutations and fusion with the phage T4 fibritin trimerisation domain [25]. WIV-1 Spike was incubated with each Fab individually and the Spike:Fab complexes were applied onto the grids. Unbound WIV-1 Spike was observed to be predominantly in a ‘closed’ conformation where all RBDs were in the ‘down’ position (S2 Fig). A high degree of Spike protein disassembly was observed on the grids of all three Fab-Spike complexes and Fabs were only observed bound to the RBD within dissociated Spikes suggesting these mAbs may have a destabilising effect on the trimer. The three mAbs bound distinct epitopes on the WIV-1 Spike, although some degree of overlap was observed in contact residues between VA14_26 and V1WT_41 (Fig 1E), which is consistent with the observed competition between VA14_26 and V1WT_41 binding for SARS-CoV-2 RBD (S1D Fig).

### Characterisation of the V1WT_06 cryptic RBD epitope

V1WT_06, the broadest and most potent mAb studied, binds to a cryptic epitope overlapping with RBD site V that is distal to the ACE2 binding site and is only accessible when RBD is in the up conformation (Fig 2A). The location of the V1WT_06 epitope is consistent with the limited competition with hu-ACE2 (Fig 1D). V1WT_06 is only minimally mutated from germline, with the heavy and light chains containing 2.8% and 1.0% nucleotide mutation from germline, respectively. Compared to other reported SARS-CoV-2 mAbs (https://opig.stats.ox.ac.uk/webapps/covabdab/), V1WT_06 harbours an unusually long CDRH3. The 30-amino acid residue CDRH3 contains two cysteine residues that form a disulfide bond, three prolines and eight aromatic amino acids. The interaction between V1WT_06 Fab and WIV-1 RBD is mediated by the heavy chain alone, with the CDRH1, CDRH2, and CDRH3 all contributing to epitope recognition (Fig 2B). In particular, S49 in CDRH1 is in close proximity to RBD residue R462, the CDRH2 loop interacts in the region surrounding the E465 patch, and the long CDRH3 likely forms interactions with the residues on the antiparallel beta strands nestled within a shallow pocket on the RBD (S4A Fig).

**Fig 2:**
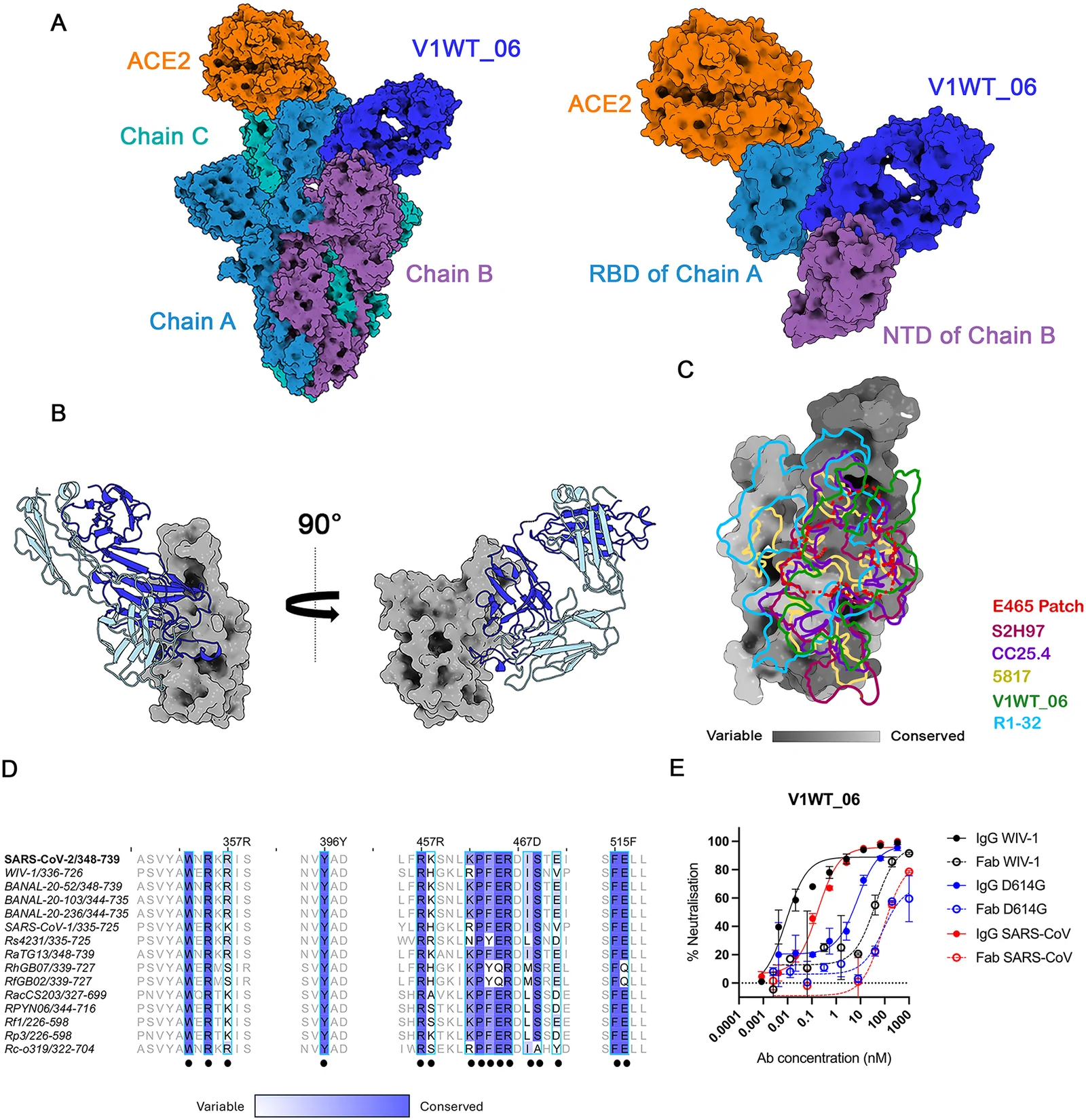
V1WT_06 binds to the RBD site V epitope using the heavy chain alone. **A)** A closeup view of the superimposed structure of V1WT_06 bound to WIV-1 RBD on a trimeric spike (from PDB: 8WLZ). A clash with NTD of chain B is observed when V1WT_06 Fab-RBD complex is aligned onto RBD in the up conformation (chain A) in trimeric WIV-1 spike protein. Notably, the binding site is inaccessible in the RBD-down conformation. **B)** V1WT_06 heavy chain only interaction with WIV-1 RBD (grey). Heavy and light chains are shown with the dark and light blue ribbons, respectively. **C)** V1WT_06 epitope footprint overlayed with epitope footprints of other site V neutralising antibodies including S2H97, CC25.4, 5817, R1-32, and E465 patch [17,27–29,35]. SARS-CoV-2 RBD (from PDB: 6m0j) is coloured based on the amino acid conservation across 87 bat sarbecoviruses [6,23,81,82]. **D)** Sequence alignment of a panel of bat sarbecovirus RBDs. Contact residues of V1WT_06 mAb on the WIV-1-RBD are indicated with black dots. The colouring scheme describes the percent identity (PID) between the sequences as implemented in Jalview 2.11.4.0. **E)** Comparison in neutralisation potency of V1WT_06 Fab and IgG against SARS-CoV-2 (D614G), SARS-CoV-1, and WIV-1. Mean values are plotted with error bars showing ± standard deviation (s.d.). The data underlying this Figure can be found in S1 Data.

Comparison of the V1WT_06 epitope with other RBD site V SARS-CoV-2 mAbs shows a similar epitope footprint to mAbs S2H97 [17], WRAIR-2063 [26], 5817 [27], and CC25.4 [28] (Fig 2C). The epitope of RBD site V mAbs is centred around the “E465 patch” (Fig 2C) which has been reported to be mutationally constrained and evolutionarily conserved [29]. S2H97, 5817, and WRAIR-2063 have shorter CDRH3 regions (11, 18, and 17 amino acids, respectively) than V1WT_06, and their light chains also engage in epitope recognition [17,26,27]. These observations indicate that an extended CDRH3 is not a specific antibody requirement for targeting of this RBD epitope, a feature observed for some HIV-1 bnAbs [30–32].

The unchanged potency of V1WT_06 against KP.2, KP.3, KP.3.1.1, LP.1, XEC, NB.1.8.1, and XFG compared to D614G is due to the conservation of contact residues within the V1WT_06 epitope (S4B Fig). Analysis of the GISAID database shows that mutations at V1WT_06 contact sites on RBD are rarely seen in the Spike of circulating viruses (S5 Fig). Indeed, mutations in the RBD site V epitope have been shown to severely impact protein expression and might suggest that there are limited escape pathways available [17]. Alignment of WIV-1 contact residues with other bat sarbecovirus Spikes show that despite broad reactivity, not all WIV-1 RBD contact residues are conserved including R357, H458, R462, F464, E465, I468, and V471 (Fig 2D), suggesting an ability to tolerate diversity within the epitope footprint which may contribute to neutralisation breadth. Furthermore, two WIV-1 contact residues differ compared to SARS-CoV-2 RBD, R462K and V471E, which may explain the reduced neutralisation potency against SARS-CoV-2 compared to WIV-1 (Fig 1B). Docking of V1WT_06 onto a recently reported structure of Rc-o319 RBD [33,34] (PDB: 9w77) predicts a similar epitope footprint (S6A Fig).

Interestingly, for other Class V nAbs the RBD cross-binding activity did not always equate to neutralisation. For example, WRAIR-2063 had no neutralising activity against WIV-1 and SHC014 despite good binding to their RBDs [17]. Additionally, S2H97 had a lower neutralisation potency against WIV-1 (~150-fold reduction) compared to V1WT_06 (S4D, S4E Fig). Despite the considerable overlap in contact residues between site V mAbs (Fig 2C), V1WT_06 and S2H97 bind RBD with differing angles of approach (S4C Fig). The majority of site V mAbs bind the epitope perpendicular to RBD when modelled onto Spike [26]. However, the heavy chain mediated binding of V1WT_06 alters the Fab approach to be more upright in relation to RBD when in the “up” conformation (Fig 2A). This altered binding angle and heavy chain interaction would likely make the site V epitope more accessible to V1WT_06 compared to other site V antibodies which may enhance neutralisation potency.

The differences in neutralisation potency between Site V mAbs may also relate to distinct mechanisms of neutralisation. S2H97 was shown to cause rapid and premature refolding of SARS-CoV-2 Spike to the post-fusion conformation and S1 shedding [17] and binding of site V mAb R1-32 to SARS-CoV-2 Spike was shown to trigger long-range allosteric Spike trimer destabilisation using hydrogen-deuterium exchange (HDX) [35]. To determine whether V1WT_06 might share a similar neutralisation mechanism to S2H97, we measured the ability of mAbs to facilitate shedding of S1 from HEK 293T cells expressing SARS-CoV-2 Spike by flow cytometry [17]. Positive control mAb (P008_067) showed a reduction in binding indicative of S1 shedding from the cell surface, whereas V1WT_06 showed no evidence of causing shedding of S1 (S7 Fig). Modelling V1WT_06 bound to SARS-CoV-2 Spike with RBD in the “up” conformation predicts a steric clash between the Fab and the NTD from the neighbouring S1 chain (Fig 2A), similar to that observed for CC.25.4 [28] and 6D6 [36]. Wedging of the V1WT_06 Fab between S1 subunits, could conformationally restrict the Spike and/or favour trimer destabilisation, as observed in our cryo-EM analysis. Indeed, the bulkier IgG showed a 1,275- and 45-fold more potent neutralisation of WIV-1 and D614G, respectively, than the Fab alone (Fig 2E) despite there being only a 6.3- and 1.3-fold increase in binding by ELISA.

Overall, V1WT_06 binds an RBD site V epitope with a novel heavy chain only mode of binding that allows for antibody approach from a more favourable binding angle, leading to high neutralisation potency and breadth.

### VA14_26 and V1WT_41 bind epitopes overlapping with RBD Class 4 mAbs

The epitopes of VA14_26 and V1WT_41 partially overlap each other and are adjacent to the ACE2 binding site (Fig 3A–3C). Their epitopes also overlap with that of CR3022 [37] and other RBD class 4 mAbs [14], including 10–40 [38] and ADI-62113 [39] (Fig 3A). VA14_26 Fab binds RBD using both the heavy and light chains with 1,427 Å^2^ buried surface area (S8A, S8B Fig). Although the cryo-EM structure does not predict a direct clash, when superimposed to the RBD, the Fab and ACE2 would come in immediate contact to achieve a backbone separation of <10 Å at several positions (Fig 3B). Thus, a direct interaction and/or allostery may help explain the observed competition between the Fab and receptor (Fig 1D). Notably, the VA14_26 Fab construct displayed 109-fold reduced neutralisation potency compared to the full IgG (S8C Fig). Thus, the mechanism of neutralisation by this bnAb may involve bivalent binding to the Spike, which could more effectively occlude the RBD from the receptor.

**Fig 3:**
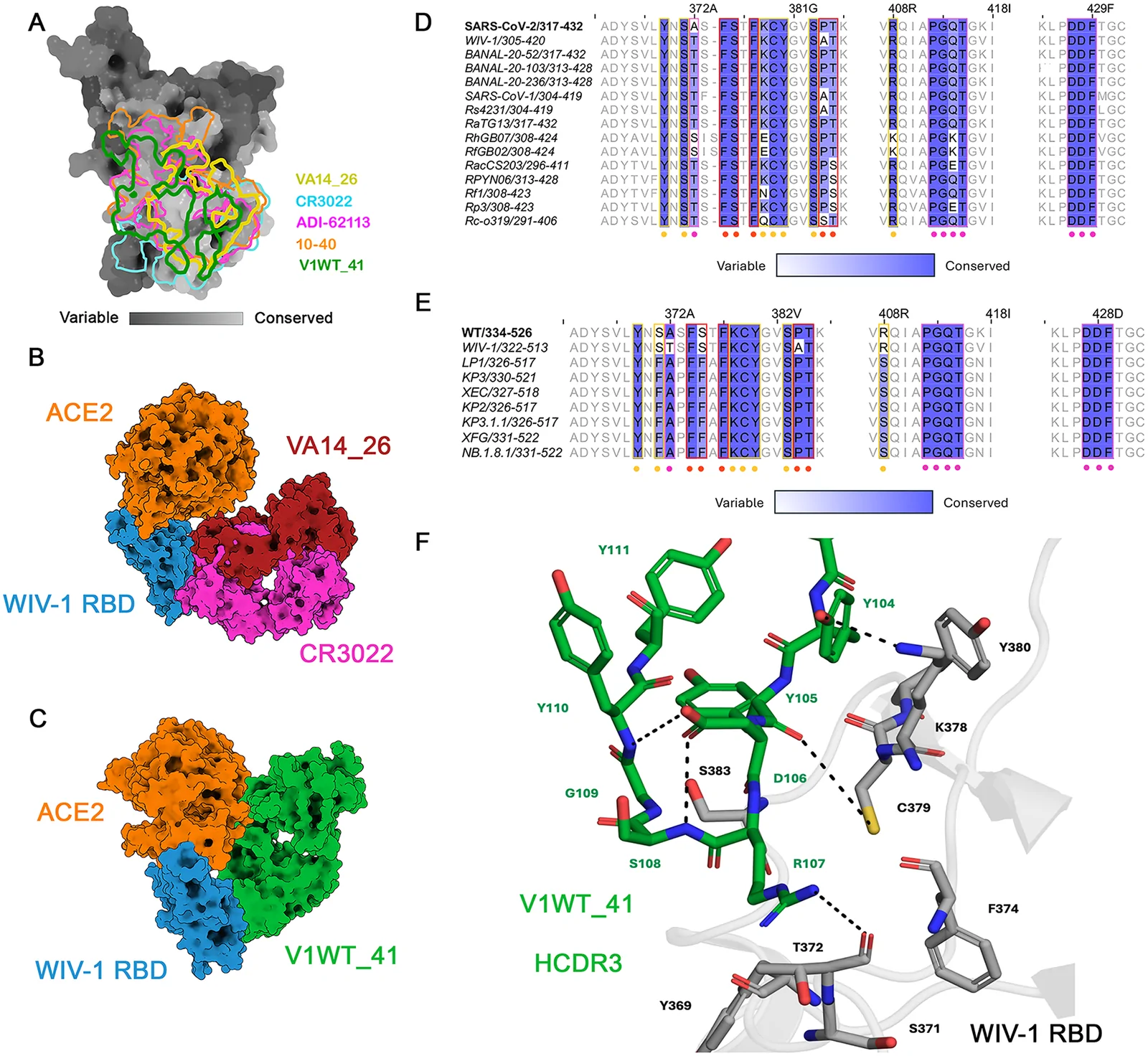
VA14_26 and V1WT_41 bind epitopes that overlap with the RBD class 4 epitope. **A)** VA14_26 and V1WT_41 antibody footprints overlayed with the epitope footprints of other RBD class 4 neutralising antibodies including CR3022, 10−40, ADI-62113 [37–39]. SARS-CoV-2 RBD (from PDB: 6m0j) is coloured based on the amino acid conservation across 87 bat sarbecoviruses [6,23,81,82]. **B)** Superimposed 3D structure depicting the binding of VA14_26 (Red) to WIV-1 RBD (blue) in relation to ACE2 (orange, from PDB: 8WLZ) and CR3022 (pink, from PDB: 6W41). **C)** Superimposed 3D structure depicting the binding of V1WT_41 (green) to WIV-1 RBD (blue) in relation to ACE2 (orange, from PDB: 8WLZ). **D)** Sequence alignment of a panel of bat sarbecovirus RBDs. Contact residues for VA14_26 and V1WT_41 are indicated by red and pink dots and outline, respectively. Shared contact residues are shown with yellow dots and outline. **E)** Sequence alignment of a panel of SARS-CoV-2 variant RBDs. Contact residues for VA14_26 and V1WT_41 are indicated by red and pink dots and outline, respectively. Shared contact residues are shown with yellow dots and outline. The colouring scheme describes the percent identity (PID) between the sequences as implemented in Jalview 2.11.4.0. **F)** A close-up view of YYDRSG motif in V1WT_41 HCDR3 (green) in contact with WIV-1 RBD (grey), shown in stick representation, renumbered based on alignment to SARS-CoV-2 [38,39]. Presence of G109 breaks the H-bonding pattern in the beta hairpin to create a G1 β-bulge. This further enables D106 side chain to make hydrogen bond with the amides in the protein backbone, thereby stabilising the local structure. Combined this accentuates a right-handed twist found in G1 β-bulge [83]. Twisting of the β-hairpin is thought to contribute towards stabilisation of RBD-Fab interaction in several other public mAbs with YYDRxG motif [38,39].

Of the three mAbs studied here, VA14_26 has the narrowest cross-reactivity with both SARS-CoV-2 variants (Fig 1B) and sarbecoviruses (Fig 1A), which is consistent with differences at critical RBD contact positions (Fig 3D, 3E). The lack of VA14_26 neutralisation against variants KP.2, KP.3, KP.3.1.1, LP.1, XEC, NB.1.8.1, and XFG is likely due to the appearance of the Spike mutations S371F, S375F, and R408S, which abolish interactions with residues in the CDRH1 and CDRH3 loops (S8A, S8B Fig). Whereas lack of binding to RhGB07 is likely due to a difference in amino acids at R378E and R408K (Fig 3D).

The epitope footprint of Fab V1WT_41 overlaps with VA14_26 but is larger in size (buried surface area of 1,795 Å^2^), extending towards the RBM and incorporating additional WIV-1 residues at P412, G413, Q414, T415, D427, D428, and F429 (Fig 3A and 3D). Although the V1WT_41 footprint does not overlap directly with the ACE2 footprint, the more downward angle of Fab binding relative to Spike when embedded upright in a membrane means there is a significant steric clash with hu-ACE2 consistent with the ACE2 competition data (Figs 3C and 1D).

The CDRH3 of V1WT_41 is 22 amino acids long and contains a YYDRxG motif that has previously been shown to be important for a subset of mAbs that recognise the Class 4 RBD site, including 10–40 [38], COVA1–16 [40], and ADI-62113 [39]. The YYDRxG motif is encoded by the D3-22 allele, an allele that is frequently used in the human antibody repertoire [38,39]. Structural analysis of ADI-62113 shows the YYDRxG motif forms a conserved structure at the tip of the CDRH3 loop that interacts with RBD and is stabilised by a preceding β-bulge [39]. The V1WT_41-RBD interface involves CDRH3 residues Y104, D106, R107, and G109 with RBD residues K378, C379, S371, and S383 (Fig 3F). The CDRH3 and framework-2 regions also interact with R408 (S8D, S8E Fig).

V1WT_41 has a broader VOC and sarbecovirus cross-reactivity than VA14_26. Although there is variation at WIV-1 contact sites for the other sarbecoviruses studied here, cross-reactivity is maintained (Figs 1A and 3D, 3E). Spike mutations S371F and R408S present in SARS-CoV-2 variants KP.2, KP.3, KP.3.1.1, LP.1, and XEC reduce neutralisation potency (25–80 -fold increase in IC_50_) but do not abolish neutralisation activity entirely (Fig 1B, 1C). The larger footprint of V1WT_41 compared to VA14_26 may allow more sequence variation to be tolerated resulting in broader cross-reactivity. In general, the V1WT_41 contact residues are very well conserved in the sarbecovirus genus (Fig 3D), suggesting that this region may have strong functional constraints that prevent Spike mutation.

Higher resolution structural analysis of Fab 10−40 (another YYDRxG mAb) with both WIV-1 and SARS-CoV-2 RBDs revealed a higher number of Fab contacts with WIV-1 compared to SARS-CoV-2 leading to higher neutralisation potency against WIV-1 [38]. Similarly, the higher neutralisation potency of V1WT-41 against WIV-1 may be related to a difference in contact residue T372A in SARS-CoV-2 (Fig 1B). Despite the disassembly of soluble WIV-1 Spike ectodomain observed for V1WT_41 and VA14_26 by cryo-EM, neither mAbs was able to facilitate shedding of the S1 from SARS-CoV-2 Spike expressed on the surface of HEK293T cells (S7 Fig).

Overall, VA14_26 and V1WT_41 bind a conserved and overlapping epitope with differing angles of approach that alters steric clash with hu-ACE2.

### Functional activity of mAbs when host cell entry is via Japanese horseshoe and Halcyon horseshoe bat ACE2

Previous studies that identified cross-reactive mAbs have generally focussed on neutralisation of sarbecoviruses entering target cells via hu-ACE2. As not all the sarbecoviruses tested utilise hu-ACE2 for cell entry [23,24], we compared the ability of mAbs to both compete with different bat species ACE2s for RBD binding and to neutralise viruses when entry was via a nonhuman ACE2, including Japanese horseshoe bat ACE2 (*Rhinolophus cornutus, Rh. cornutus*-ACE2) and Halcyon horseshoe bat ACE2 (*Rhinolophus alcyone, Rh. alcyone*-ACE2) (Fig 4).

**Fig 4:**
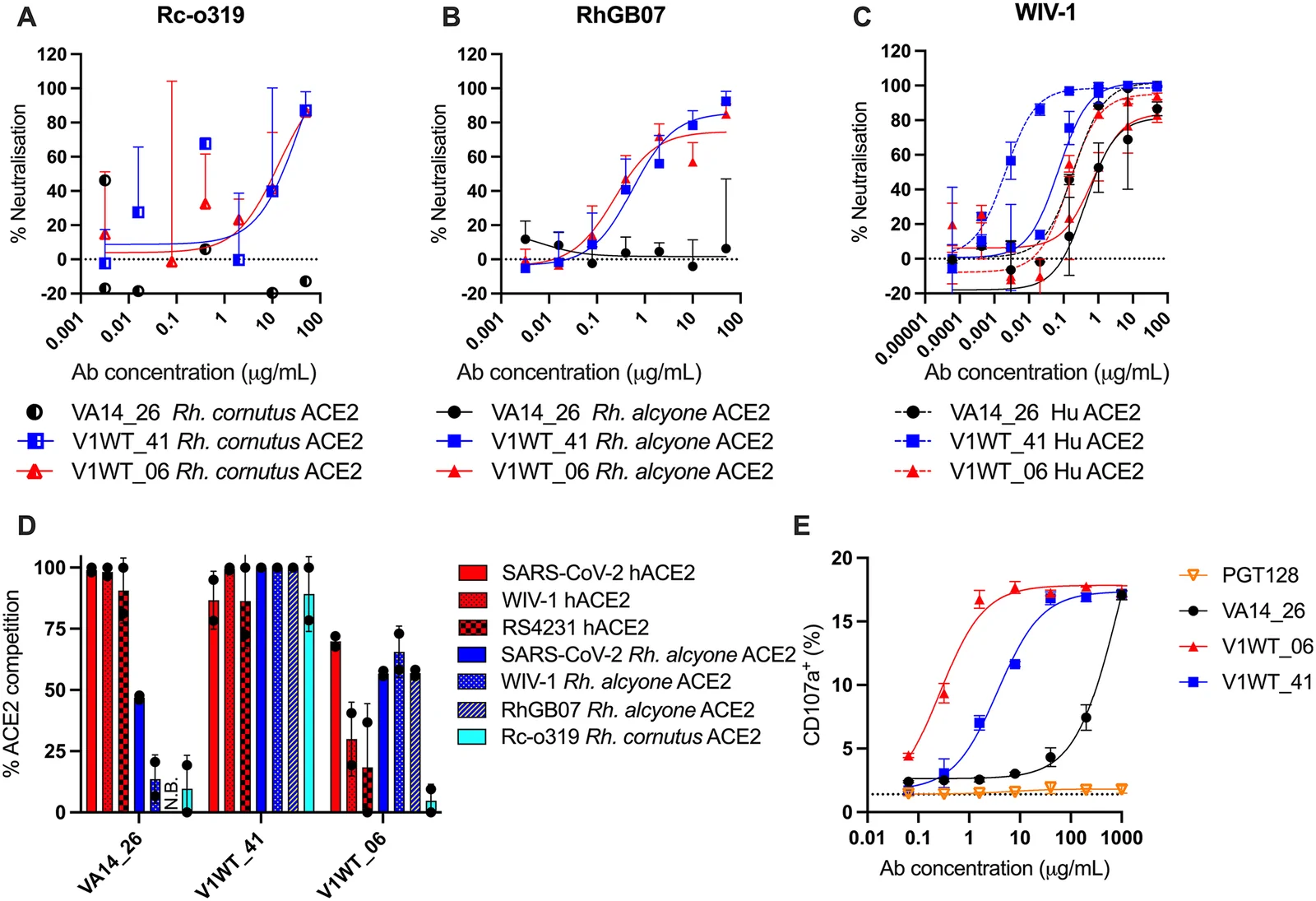
Mechanism and functional activity of V1WT_06, V1WT_41, and VA14_26. Neutralisation of sarbecoviruses through entry via different host ACE2s. Neutralisation of **A)** Rc-o319 and **B)** RhGB07 was measured against entry via *Rh. cornutus* ACE2 and *Rh. alcyone* ACE2, respectively, which were transiently expressed in HEK293T/17 cells. **C)** Neutralisation of WIV-1 was compared between entry via hu-ACE2 (dotted line and closed symbols) and *Rh. alcyone* ACE2 (full line and closed symbols). Neutralisation was measured using HEK 293T cells transiently expressing either *Human* ACE2 or *Rh. alcyone* ACE2. For A-C, mean values are plotted with error bars showing ± standard deviation (s.d.). **D)** Ability of IgGs to inhibit the interaction between cell surface ACE2 (including hu-ACE2, *Rh. cornutus* ACE2 and *Rh. alcyone* ACE2) and soluble RBD (including SARS-CoV-2, WIV-1, Rs4231, RhBG07, and Rc-o319 RBDs). IgGs were pre-incubated with fluorescently labelled RBD before addition to ACE2 expressing cells. N.B = No binding of IgG to the RBD. The percentage reduction in mean fluorescence intensity is reported. Experiments were performed in duplicate. **E)** Antibody dependant cellular cytotoxicity (ADCC) of IgGs measured through the surface expression of CD107a on activated NK cells (NK-92 cells expressing CD16) [41]. HIV-1 specific neutralising antibody, PGT128 is used as a negative control [31]. The data underlying this Figure can be found in S1 Data.

First, we measured the ability of the three mAbs to neutralise RhGB07 and Rc-o319 entering via *Rh. alcyone* ACE2 and *Rh. cornutus* ACE2, respectively. Consistent with the cross-binding reactivity of mAbs (Fig 1A), V1WT_06 and V1WT_41 were able to neutralise RhGB07 and showed very modest neutralisation of Rc-o319, whilst VA14_26 showed no neutralisation against either virus (Fig 4A, 4B). Next, we compared neutralisation of WIV-1 when cell entry was mediated by *Rh. alcyone* ACE2 or hu-ACE2. The neutralisation of WIV-1 by V1WT_06 and VA14_26 appeared independent of the host origin of the ACE2 receptor used, whereas V1WT_41 was more potent at neutralising WIV-1 when entry was mediated by hu-ACE2 (Fig 4C).

To complement the neutralisation studies, we compared the ability of mAbs to inhibit binding of SARS-CoV-2, WIV-1, Rc-o319 RBDs to *Rh. cornutus* ACE2 [33], and binding of RhGB07 RBD to *Rh. alcyone* ACE2. As expected, there was minimal ACE2 competition for site V binding mAb V1WT_06. V1WT_41 competed with ACE2 binding for all RBD and ACE2 pairings (Fig 4D), confirming that inhibition of ACE2 binding is the likely mechanism of V1WT_41 neutralisation. The V1WT_41 competition with *Rh. cornutus* ACE2 is consistent with a clash between V1WT_41 Fab and *Rh. cornutus* ACE2 when docked onto the Rc-o319 RBD structure [33,34] (S6C Fig). However, the more potent V1WT_41 neutralisation of WIV-1 entering through hu-ACE2 does not appear to be due to more efficient ACE2 competition. In contrast, despite VA14_26 showing potent neutralisation of WIV-1 entry via both hu-ACE2 and *Rh. alcyone*-ACE2, VA14_26 showed only partial competition with *Rh. alcyone*-ACE2 binding for WIV-1 and SARS-CoV-2 (Figs 4D and S6B) suggesting the mechanism of VA14_26 neutralisation is only partially dependent on ACE2 competition. Overall, these data show that neutralisation breadth is conserved for different sarbecovirus and host ACE2 combinations.

### mAbs show potent ADCC activity

Finally, we determined whether these mAbs could facilitate antibody-dependent cellular cytotoxicity (ADCC) against ancestral SARS-CoV-2 [41,42]. All three cross-reactive mAbs demonstrated Wuhan-1 specific potent degranulation of an NK cell line expressing CD16 (NK-92) detected through measurement of CD107a expression by flow cytometry (Fig 4E) whereas the negative control, HIV-specific mAb PGT128, showed no activity. VA14_26 had the lowest activity which is consistent with the lower binding affinity to Spike. Interestingly, mAbs 5817 and S2H97, which bind an epitope overlapping with V1WT_06 were reported not to mediate potent ADCC activity [17,43], suggesting the angle of antibody binding can also play a role in effector function activity. Overall, in a scenario where cross-sarbecovirus binding exists in the absence of neutralisation, effector functions of mAbs generated following SARS-CoV-2 infection/vaccination could still provide some level of protection upon viral exposure [42].

## Discussion

Here we describe the neutralising epitopes of three novel mAbs that have broad SARS-CoV-2 variant and sarbecovirus cross-binding and cross-neutralisation. The continued evolution of SARS-CoV-2 Spike and the sustained threat of novel sarbecoviruses zoonosis into the human population warrants a more complete understanding of neutralising epitopes that are highly conserved across sarbecoviruses. Identification of broad antibodies and conserved epitopes will have application to development of next generation antibody-based therapeutics and for rational vaccine design.

mAbs with a high degree ofsarbecovirus cross-reactivity appear to be relatively rare within the immune repertoire of SARS-CoV-2 infected and/or vaccinated individuals but several broad nAbs targeting RBD have been reported previously [17,44–46]. We report three mAbs with broad sarbecovirus activity which were isolated from donors with minimal and differing SARS-CoV-2 exposure histories. Only two AZD1222 vaccinations were required to elicit VA14_26 [22], whilst V1WT_41 and V1WT_06 were isolated from a donor that received two BNT162b2 vaccinations and experienced a breakthrough infection with delta VOC [8]. Other studies reporting cross-reactive mAbs to RBD class 4 and site V were also isolated from individuals with minimal SARS-CoV-2 exposures [13,14,17,47]. This observation differs from HIV-1 infection where chronic antigen stimulation and extensive somatic hypermutation are required to achieve neutralisation breadth against multiple HIV-1 strains [30,48].

While the RBM is highly variable between sarbecoviruses and SARS-CoV-2 variants, the RBD does harbour conserved epitopes. RBD site V is highly conserved across sarbecoviruses, and deep mutational scanning has shown that mutation around the E465 patch severely impacts on RBD expression [29], highlighting the evolutionary and functional constraints on the site V epitope. V1WT_06 is unique compared to other site V nAbs as binding is mediated by the heavy chain alone. This property enables the mAb to approach the cryptic site at an oblique angle compared to other site V nAbs, potentially permitting access to a transitional state of Spike with relatively narrow opening between the NTD and RBD on neighbouring protomers. HIV-1 broadly neutralising antibodies (bnAbs) that target the V2-apex bnAb epitope are also dominated by heavy chain interactions where a long CDRH3 penetrates through the glycan shield to contact conserved residues at the base of the glycans [30,49]. However, the light chain also makes minor contacts with HIV-1 Env [32] but there is flexibility in terms of light chain pairing [50]. It may be possible to adapt vaccination strategies engineered to selectively elicit HIV-1 antibodies with long CDRH3s to engineer immunogens that elicit V1WT_06-like nAbs against the RBD site V epitope [51–53].

The mechanism of virus neutralisation by mAbs to site V is not fully understood. Whilst mAbs such as S2H97 are able to facilitate S1 shedding [17], not all sarbecoviruses Spikes encode an S1/S2 furin cleavage site, which is conceivably required for physical loss of the S1 subunit [54]. However, our data suggests that binding of non-neutralising antibodies to the site V epitope may still facilitate effector functions such as ADCC which are important for protection from infection [42]. V1WT_06 was able to neutralise all sarbeoviruses tested regardless of the S1/S2 furin cleavage site indicating an alternative neutralisation mechanism to S1 shedding. Modelling of the V1WT_06/RBD bound to trimeric Spike suggests Fab binding drives a wedge between RBD and NTD on neighbouring protomers that may destabilise the Spike protein leading to the Spike disassembly observed in our cryo-EM data, and similar to the destabilisation mechanism observed for site V mAb R1-32 [35].

The remarkable conservation of the RBD class 4 site is thought to be due to its role in stabilising the RBD-RBD interface when RBD is in the down conformation and it can also form contacts to the S2 domain [37]. The limited exposure of this epitope and the subsequent low frequency of mAbs targeting this domain might also reduce the immune selective pressure on this epitope leading to limited variation in this region. V1WT_41 belongs to the well-described public antibody class encoding a YYDRxG motif in the CDRH3 region [13,38,39]. The mechanism of V1WT_41 neutralisation is through sterically restricting ACE2 binding, a mechanism we show is conserved when sarbecovirus entry is mediated through ACE2 of Japanese (*Rh. cornutus*) and Halcyon (*Rh. alcyone*) horseshoe bats.

The VA14_26 epitope overlaps with the CR3022 epitope but is considerably broader in neutralisation than CR3022 [22,37]. Broadly reactive mAbs that target the CR3022 epitope are rare. Examples include C118 which achieves cross-neutralisation through a long CDRH3 reaching the conserved patch on RBD as well as contacting backbone residues in RBD allowing tolerance of some degree of variation [55]. The mechanism of VA14_26 neutralisation is less clear than V1WT_41 as the more perpendicular angle of Fab binding and the epitope position suggests that the steric clash with hu-ACE2 is minimal. Furthermore, VA14_26 shows limited competition with *Rh. alcyone*-ACE2 for WIV-1 RBD binding compared to hu-ACE2 but showed similar neutralisation potency when cell entry was mediated by *Rh. alcyone*-ACE2 or hu-ACE2. As VA14_26 showed no evidence of causing S1 shedding, the mechanism of neutralisation is likely through Spike destabilisation.

The low frequency of mAbs to RBD class 4 and site V is likely explained by the transient exposure of their epitopes only when RBD is in the “up” conformation. By contrast, the immunodominant neutralising epitopes on RBD map to the RBM (including Class 1 and 2 epitopes) [13,14,17]. Antibodies targeting these epitopes tend to be highly potent, but their epitopes are more highly mutated, particularly in SARS-CoV-2 variants, due to the selective pressure of nAbs leading to high sequence variation and minimal breadth. The key challenge for rational immunogen design is to engineer immunogens that selectively expose or unmask these subdominant yet highly conserved class 4 and site V RBD epitopes. When considering these epitopes in the context of sarbecoviruses more broadly, the WIV-1 Spike is mostly found with RBD in the “down” conformation [56], suggesting immunogens based on the full Spike protein will be poor at inducing such antibodies. Several groups have investigated the use of mosaic RBD nanoparticle vaccines for elicitation of broadly reactive RBD-binding antibodies in mice. The multivalent presentation of mixed RBDs was shown to preferentially generate antibodies towards the conserved Class 1/4 [57] and site V epitopes [58]. In particular, a self-assembling lumazine synthase (LuS) scaffold displaying clade 1a, 1b, and 2 RBDs led to a dominant response towards an epitope that overlapped with that of S2H97 [58]. It is thought that multivalent presentation of mixed nanoparticles preferentially expands B cells with cross-reactive BCRs. This approach appears a promising strategy for eliciting nAbs to these conserved but minimally exposed RBD site V and class 4 epitopes.

In summary, the novel V1WT_06, V1WT_41 and VA14_26 mAbs are candidates for development of antibody-based therapeutics against newly emerging sarbecoviruses and can provide templates for rational development of pan-sarbecovirus vaccines.

## Materials and methods

### Monoclonal antibody isolation and expression

VA14_26 [22], and V1WT_06 and V1WT_41 [8] were isolated previously. IgG1 antibody heavy and light plasmids were co-transfected at a 1:1 ratio into HEK-293F cells (Thermofisher) using PEI Max (1 mg/mL, Polysciences) at a 3:1 ratio (PEI Max:DNA). Antibody supernatants were harvested five days following transfection, filtered, and purified using protein G affinity chromatography following the manufacturer’s protocol (GE Healthcare).

### RBD expression and purification

Recombinant RBD of SARS-CoV-2, WIV-1, Rs4231, RacCS203, Rc-o319, RhGB07 for ELISA were expressed and purified as previously described [20]. Briefly, RBDs were cloned into the pOPIN-BAP-His expression vector in Expi293F cells, transfected using PEI40K (Polysciences) with additives (300 mM valproic acid, 500 mM sodium propionate, 2.5 M glucose. Supernatants were harvested 4 days post-transfection and presence of secreted protein was determined by Coomassie and by blotting for anti-His. Supernatants were purified using HisTrapHP columns (Cytivia), desalted using Zeba Spin Desalting Columns (ThermoFisher Scientific) and concentrated using Amicon Ultra 15 columns (Merck Millipore), before quantification by Nanodrop and a Pierce BCA Protein Assay (ThermoFisher Scientific).

### WIV-1 Spike cloning, expression and purification

For cryo-EM, trimeric WIV-1 spike ectodomain (corresponding to residues 1-1191, GenBank AGZ48831.1) with amino acid substitutions for stabilising the pre-fusion conformation (K969P, V970P) and K668S followed by carboxy-terminal foldon and hexahistidine tag [25] was expressed in HEK-293F cells and purified as previously described [59].

### Fab cloning and expression

The variable heavy and light chain domains were cloned into the pHLsec vector [60] containing the CH1 or CK1 domains, respectively. Antibody heavy and light plasmids were co-transfected at a 1:1 ratio into HEK-293F cells (Thermofisher) using PEI Max (1 mg/mL, Polysciences) at a 3:1 ratio (PEI Max:DNA). Antibody supernatants were harvested five days following transfection, filtered and purified using immobilised metal affinity chromatography and size-exclusion chromatography.

### ELISA binding

96-well plates (Corning, 3690) were coated with RBD antigen at 3 μg/mL overnight at 4 °C. The plates were washed (5 times with PBS/0.05% Tween-20, PBS-T), blocked with blocking buffer (5% skimmed milk in PBS-T) for 1 h at room temperature. Serial dilutions of IgG or Fab in blocking buffer were added and incubated for 2 h at room temperature. Plates were washed (5 times with PBS-T) and secondary antibody was added and incubated for 1 h at room temperature. IgG was detected using Goat-anti-human-Fab-AP (alkaline phosphatase) (1:1,000) (Jackson: 109-055-006). Plates were washed (5 times with PBS-T) and developed with AP substrate (Sigma) and read at 405 nm. Graphs were generated using GraphPad Prism version 10.

### Pseudotyped virus production

Pseudotyped HIV-1 virus incorporating each of the SARS-CoV-2 variants (D614G, KP.2, LP.1, KP.3, KP.3.1.1, and XEC) and sarbecovirus Spikes (SARS-CoV, WIV-1, RhGB07, and Rc-o319) were generated by first seeding HEK293T/17 cells in 10 cm dishes at a density of 3 x 10^5^ cells/mL in Dulbecco’s Modified Eagle Medium (DMEM) with 10% foetal bovine serum (FBS), 100 U/mL penicillin and 100 µg/mL streptomycin. Following overnight culture, cells were co-transfected using 90 µg PEI-Max (1 mg/mL, Polysciences) with 15 µg HIV-luciferase plasmid, 10 µg HIV 8.91 gag/pol plasmid, and 5 µg Spike protein plasmid [61]. Transfected cells were incubated for 72 h at 37 °C, and virus was harvested, sterile filtered, and stored at −80 °C until required.

### Neutralisation assays

Neutralisation assays were conducted as previously described [62–64] using either HeLa cells stably expressing the ACE2 receptor (provided by Dr James Voss, Scripps Research, La Jolla, CA) or HEK 293T cells transiently transfected with either Human (provided by Prof Michael Malim, KCL), Halcyon horseshoe bat (*Rh. alcyone*) or Japanese horseshoe bat (*Rh. cornutus*) ACE2. HEK293T cells were transfected with either Human, Halcyon or Japanese horseshoe bat ACE2 using PEI Max (1 mg/mL, Polysciences) at 3:1, 6:1, and 2:1 ratio (PEI Max:DNA), respectively. Cells were harvested after 72 h using EDTA.

Serial dilutions of IgG or Fab were prepared with DMEM-C media (10% Fetal Calf Serum and 1% Penicillin/Streptomycin) and incubated with pseudotyped virus for 1 h at 37 °C in 96-well plates. Next, HeLa-ACE2 cells (12,500 cells/50 µL per well) or transfected HEK293T/17 cells (15,000 cells/25 µL per well) were added and the plates were left for 72 h. The amount of infection was assessed in lysed cells with the Bright-Glo luciferase kit (Promega), using a Victor X3 multilabel reader (Perkin Elmer). Measurements were performed in duplicate and duplicates used to calculate the ID_50_. Graphs were generated using GraphPad Prism version 10.

### ACE2 competition measured by flow cytometry

Purified mAbs were mixed with His tagged RBD in a molar ratio of 4:1 on ice for 1 h. HeLa-ACE2 cells or HEK293T/17 cells transiently expressing *Rh. alcyone*-ACE2 or *Rh. Cornutus*-ACE2 were washed once with PBS and detached using PBS containing 5 mM EDTA. Detached cells were washed and resuspended in FACS buffer. Cells (0.5 million) were added to each mAb-RBD complex and incubated on ice for 1 h. RBDs were stained concomitantly, using FITC-conjugated Rabbit-anti-HIS antibody (Abcam, Ab1206) at 5 μg/ml for a final volume of 100 μl. The cells were washed with PBS and resuspended in 1 mL FACS buffer with LIVE/DEAD Fixable Aqua Dead Cell Stain Kit (1 μL, Invitrogen). Cells only and cells with RBD only were used as background and positive controls, respectively. The geometric mean fluorescence of FITC was measured from the gate of singlet cells as previously described [65]. ACE2 binding inhibition was calculated with this equation:


$$\begin{array}{c} \%\;ACE\text{2}\;binding\;inhibition=\text{1}\text{0}\text{0}*\left(\text{1}-\frac{\;sample\;geometric\;mean-geomatric\;mean\;of\;background}{geometric\;mean\;of\;positive\;control-geometric\;mean\;of\;background}\right) \end{array}$$

### S1 shedding assay

HEK293T/17 cells were plated in a 6-well plate (2 x 10^6^ cells/well). Cells were transfected with 1 μg of plasmid encoding full-length SARS-CoV-2 Spike using PEI Max 40K (3 μL at 1 mg/mL) and incubated for 48 h at 37 °C. Post incubation cells were resuspended in PBS and plated in U-bottom 96-well plates (1 x 10^5^ cells/well). Monoclonal antibodies were diluted in FACS buffer (1x PBS, 2% FBS, 1 mM EDTA) to 25 μg/ml and incubated with cells on ice for 5, 10, 20, 40, or 80 mins. The plates were washed twice in FACS buffer and stained with 50 μl/well of 1:200 dilution of PE-conjugated mouse anti-human IgG Fc antibody (BioLegend) on ice in dark for 1 h. After another two washes, stained cells were analysed using flow cytometry, and the binding data were generated by calculating the percent (%) PE-positive cells using FlowJo 10 software. Mean fluorescence intensity (MFI) for each sample was determined at each time point and each sample was normalised to the MFI at the 5 min time point (MFI/MFI at 5 min × 100).

### Binding to cell surface expressed Spike

HEK293T/17 cells were plated in a 10 cm dish at a seeding density of 5 x 10^5^ cells/ml. Cells were transfected with 10 μg of plasmid encoding full-length Spike from a range of sarbecoviruses using PEI Max 40K (30 mL at 1 mg/mL) and incubated for 48 h at 37 °C. WIV-1 full spike was used as a positive control. Post incubation, cells were resuspended in PBS and plated in V-bottom 96-well plates (3 x 10^5^ cells/well). On separate occasions, monoclonal antibodies were diluted in FACS buffer (1x PBS, 2% FBS, 1 mM EDTA) to 10 μg/ml and incubated with cells on ice at a volume of 100 μl for 30 mins. The plates were washed twice in FACS buffer and stained with 100 μl/well of APC-conjugated mouse anti-human IgG Fc antibody (1:333 dilution, BioLegend) on ice in dark for 1 h. After another two washes, stained cells were analysed using flow cytometry, and the geometric mean fluorescence intensity (GMFI) was calculated by gating out the untransfected cells using FlowJo 10 software. Expression of FLAG-tagged Spikes was confirmed by western blot (F3165, Sigma Aldrich).

### ADCC assay

NK-92 cells expressing human CD16 were maintained in Minimum Essential Medium-α (MEM-α) supplemented with L-glutamine, nucleosides, 12.5% Foetal Calf Serum, 12.5% Horse Serum, 20 mM HEPES, 0.2 mM Myo-inositol, 0.02 mM Folic Acid, 0.1 mM 2-mercaptoethanol and 50 IU/ml Interleukin-2 (IL-2).

High-binding ELISA plates were coated with recombinant WT Spike protein at 3 μg/mL in PBS for 2 h at 37 °C. Wells were washed with PBS and then blocked with 5% bovine serum albumin in PBS overnight at 4 °C. The plates were washed with PBS, prior to addition of monoclonal antibodies diluted in fully supplemented MEM-α and incubated at room temperature for 1 h. NK-92 CD16 cells were added (1.0 × 10^5^ cells/well) in media supplemented with 100 IU/ml IL-2, Golgistop protein transport inhibitor (BD Biosciences #554724) and PerCP/Cyanine5.5 anti-human CD107a antibody (Biolegend Clone H4A3). Plates were incubated at 37 °C, 5% CO_2_ for 6 h. NK-92 CD16 cells were transferred to 75 mm Polystyrene Tubes, washed with PBS and fixed by addition of 4% paraformaldehyde. Level of CD107a surface expression was measured by flow cytometry using a BD FACSCanto II. Graphs were generated using GraphPad Prism version 10.

### Cryo-EM data collection, image processing, and structure refinement

WIV-1 Spike ectodomain (3.5 μl, 1.3 mg/mL), supplemented with V1WT_41, VA14_26, or V1WT_06 Fab (0.2 mg/mL, corresponding to a 1.3-fold molar excess to Spike) in 150 mM NaCl, 0.1% n-octyl glucoside, and 20 mM Tris-HCl, pH 8.0, was applied to 300-mesh 1.2/1.3 C-flat holey carbon grids (Electron Microscopy Sciences) for 1 min under 95% humidity at 20 °C and blotted for 3−6 s, and the samples were vitrified by plunge-freezing in liquid ethane/propane using a Vitrobot Mark IV (Thermo Fisher Scientific). Cryo-EM data were acquired across on Titan Krios cryo-electron microscopes with Falcon 4i direct electron detectors (Thermo Fisher Scientific; V1WT_41 and VA14_26). For imaging of the V1WT_06 Fab-Spike complex, the microscope was additionally equipped with a Selectris energy filter with an energy slit set to 10 eV. Micrograph movie stacks (4,500 for V1WT_41 and VA14_26 and 7,058 for V1WT_06 FAB-Spike complex) were recorded in the dose-fractionation mode, at a calibrated magnification corresponding to 1.08 Å (V1WT_41 and VA14_26) or 0.95 Å (V1WT_06) per physical pixel and a total sample exposure dose of 32.2 e/Å^2^ (V1WT_41 and VA14_26) or 41.0 e/Å^2^ (V1WT_06). A defocus range of −1.5 μm to −3.3 μm was used for data collection (S1 Table). 1,674 EER frames, recorded per micrograph movie, were subsequently processed in 31 fractions.

The micrograph stacks were aligned and summed, with dose weighting applied, as implemented in MotionCor2 [66]. Contrast transfer function (CTF) parameters were estimated from image sums using GCTF v1.18 [67]. Initial particle picking was done using crYOLO with general model (gmodel_phosnet_202005_N63_c17.h5) [68]. The resulting 730,359 (VA14_26), 576,253 (V1WT_41), and 1,011,931 (V1WT_06) particles were extracted in RELION- 4.0 [69] binned 4-fold to a pixel size of 4.32 Å (V1WT_41 and VA14_26) or 3.8 Å (V1WT_06). These were subjected to four rounds of reference-free 2D classification in cryoSPARC v4.6.2 [70]. Initially, particles belonging to well-defined 2D classes corresponding for the Spike trimer were selected and subjected to ab-initio reconstruction followed by heterogeneous and nonuniform refinement in cryoSPARC (S2 Fig). Neither the 2D nor 3D class averages of the trimeric spike revealed features attributable to a bound FAB molecule (S2 Fig). By contrast, FAB-like features were readily identifiable in 2D class averages corresponding to dissociated Spike (S2 Fig).

Particles contributing to 2D classes of the dissociated Spike were subjected to ab-initio reconstruction followed by heterogeneous refinement in cryoSPARC with 4 classes. Those belonging to the most populated well-resolved 3D classes were used to train a model for picking the entire datasets using Topaz [71]. Newly identified particles extracted binned 4-fold were subjected to three rounds of 2D classification and those contributing to well-defined 2D averages were used for ab-initio reconstruction and, subsequently, heterogenous refinement. The remaining particles were re-extracted with 2-fold binning and subjected to ab-initio reconstruction (5 classes), the obtained reconstructions revealed well-defined 3D reconstructions, readily interpretable as S1 subunit associated with Fab moieties (S2 Fig). Following 3 iterations of heterogeneous refinement in cryoSPARC, 142,232 (VA14_26-S1 complex), 256,418 (V1WT_41-S1 complex), and 135,592 (V1WT_06-S1 complex) particles were re-extracted without binning. These particles were then subjected to ab-initio reconstruction with 5 classes followed by heterogenous refinement in cryoSPARC. Particles from the most populated classes were subjected to Bayesian polishing in Relion-5.0 [72]. Next, ab-initio reconstruction was performed with the polished particles with class similarity set to 0 and number of classes set to 3 (S2 Fig). Subsequently, heterogeneous refinement was conducted using the volume data derived from the ab-initio reconstructions. Local CTF refinement allowed to improve quality of VA14_26-S1 and V1WT_41-S1 maps. The final reconstructions were obtained using nonuniform refinement in cryoSPARC (S1 Table, S2 Fig). The resolution metrics reported here are according to the gold-standard Fourier shell correlation (FSC) 0.143 criterion [73]. Local resolution was estimated in cryoSPARC (S3 Fig). The atomistic model for monomeric S1 protein was derived from PDB entry 8TC0. Antibodies were modelled for their translated amino acid sequence using AlphaFold 3 server (https://alphafoldserver.com/welcome) [74]. Models were docked into cryo-EM maps using UCSF Chimaera [75]. Guided by the cryo-EM maps, the models were fitted interactively in Coot-0.9.8.95 [76]. The model was refined using CryoSPARC maps filtered according to local resolution metrics in phenix.real_space_refine (version dev-4213) [77,78]. The quality of the final model was assessed using MolProbity [79]. The final cryo-EM reconstruction and refined model have been deposited with the EMDB and the PDB under accession codes 9S6Y, 9S6Q, and 9S67.

### Analysis of Spike mutations in circulating SARS-CoV-2

The complete SARS-CoV-2 metadata file was downloaded from the GISAID [80] EpiCoV database (https://gisaid.org) on 30/04/2025; details on these sequences are available at https://doi.org/10.55876/gis8.250704no. The file contains an entry for each SARS-CoV-2 sequence, including sample information (host, collection date, geographical location, etc) as well as SARS-CoV-2 genome sequence information (length, pangolin lineage, nonsynonymous mutations in each ORF, etc). A python script was written to parse this file and extract and count all nonsynonymous mutations observed across all Spike amino acid positions; sequences that were not human, were less than 20,000 nucleotides, had an unassigned pangolin lineage or were labelled as “low coverage” were excluded, leaving 15.6 million sequences.

### Surface Plasmon Resonance

Kinetics and epitope binning assays were performed on the Carterra LSA^XT^ using HC30M chips as previously described [20]. Briefly, chips were pre-conditioned using sequential injections of NaOH, NaCl, and glycine (pH 2.5). For kinetics, an anti-human Fc lawn was covalently coupled to the HC30M surface using EDC/NHS chemistry. V1WT_06, V1WT_41, and VA14_26 were diluted in PBST (PBS + 0.005% tween 20) to 1 μg/mL and 0.25 μg/mL and captured to the anti-human-Fc lawn for 7 min. Single cycle (nonregenerative) kinetics were performed using RBD diluted 2-fold in PBST (5 μg/mL to 2.4 ng/mL) allowing a 7-min association and 14-min dissociation. Surface regeneration was achieved using glycine (pH 1.9, 10 mM). Kinetic analysis was performed in Carterra Kinetics software v1.9.2 to calculate Ka, Kd, and KD. Data were fitted using 2000 iterations with a minimum allowable Kd of 2.5E-05. Average Ka, Kd, and KD were calculated using the values obtained from both coupling concentrations and the two independent couplings on the chip giving *n* = 4. For epitope binning, human anti-RBD monoclonal antibodies were directly covalently coupled to the HC30M surface. SARS-CoV-2 wild type RBD was injected first followed by the competing antibody. Surface regeneration was achieved as above. Epitope binning analysis was performed in Carterra Epitope software v1.9.2.

## Supporting information

S1 Fig Antibody binding affinity and kinetics and antibody binding competition. **A)** Affinity (K_D_), association rate constant (k_a_) and dissociation rate constant (k_d_) of mAb binding kinetics for SARS-CoV-2 RBD were measured by surface plasmon resonance using the Carterra LSA platform. Values are coloured in accordance with the gradients presented. **B)** Binding of mAbs to cell surface expressed Spike by flow cytometry including Clade 1a (Rs4231, WIV-1), Clade 1b (RpYN06) and Clade 2 (RacCS203, RP3/2004, and RF1/2004). **C)** Each Spike had a C-terminal FLAG-tag and expression of Spike was confirmed by western blot of transfected cell lysates. **D)** Heatmap of antibody blocking and sandwiching interactions between VA14_26, V1WT_41, and V1WT_06 measured using SPR against SARS-CoV-2 RBD. Antibodies immobilised as ligands are listed on the left and analytes injected are listed across the top. The scale shows the normalised change in response units (RU) relative to injection of PBS after RBD. RU > 0.3 show sandwiching analytes (i.e., no competition) and RU < 0.3 shows blocked or displaced analytes (i.e., competition). The data underlying this Figure can be found in S1 Data.
(TIF)

S2 Fig Representative workflow for V1WT_41 Fab-spike complex dataset. Representative micrograph shown (scale bar at 50 nm). Representative 2D class average corresponding to Spike trimer ectodomain is displayed. Nonuniform refinement of the WIV-1 ectodomain trimer reconstruction presented next to viewing direction distribution plot for all three datasets. Notably features corresponding to a bound Fab molecule are absent in 2D and 3D classes. Representative 2D class averages corresponding to dissociated spike protomer bound to V1WT_41 Fab is shown. Panels include Half-map Fourier shell correlation curve (FSC curve) and viewing direction distribution plot (cryoSparc) for final reconstruction of Fab-Spike protomer complexes. Abbreviation: Ptcls – Particles. Related to S3 Fig.
(TIF)

S3 Fig Map to model fit and estimated local resolution for final 3D reconstructions of Fabs bound to WIV1 spike protomer. **A)** Panel displaying the overall map to model fit for V1WT_06 Fab (red) bound to WIV1 spike (blue). The insets show a zoomed in view of the Fab-spike interface. The reconstruction is coloured by estimated local resolution (cryoSparc) overlayed on B-factor sharpened maps, colour scheme provided on the right. **B)** Panel displaying the overall map to model fit for V1WT_41 Fab (red) bound to WIV1 spike (blue). The insets show a zoomed in view of the Fab-spike interface. The reconstruction is coloured by estimated local resolution (cryoSparc) overlayed on B-factor sharpened maps, colour scheme provided on the right. **C)** Panel displaying the overall map to model fit for VA14_26 Fab (red) bound to WIV1 spike (blue). The insets show a zoomed in view of the Fab-spike interface. The reconstruction is coloured by estimated local resolution (cryoSparc) overlayed on B-factor sharpened maps, colour scheme provided on the right.
(TIF)

S4 Fig Binding of V1WT_06 to WIV-1 RBD. **A)** Closeup view of V1WT_06 HCDRs and the WIV-1 RBD interface. Individual CDRH1, CDRH2, and CDRH3 views have been shown with contact residues on the RBD shown as sticks and numbered based on WIV-1. **B)** Sequence alignment of a panel of SARS-CoV-2 Variant RBDs. Contact residues of V1WT_06 mAb on the WIV-1-RBD are indicated with black outline. The colouring scheme describes the percent identity (PID) between the sequences as implemented in Jalview 2.11.4.0. **C)** Superimposed 3D structure of S2H97 (lime green), another RBD site V neutralising antibody (from PDB: 7M7W) and ACE2 (orange, from PDB: 8WLZ) shown around WIV-1 RBD – V1WT_06 (dark blue) Fab complex. Angle of approach of V1WT_06 in relation to S2H97 contrasts the RBM proximal, heavy chain driven interaction of V1WT_06 as opposed to RBM distal angle of approach of S2H97 [17]. **D)** and **E)** Neutralisation potency of Site V mAb S2H97 compared to V1WT_06. Mean values are plotted with error bars showing ± standard deviation (s.d.). The data underlying this Figure can be found in S1 Data.
(TIF)

S5 Fig Conservation of mAb contact residues observed in the GISAID data base. Frequency of mutations at RBD contact residues observed in the GISAID database. Residue position is based on SARS-CoV-2 numbering. Contact residues are coloured according to the mAb; V1WT_06 (blue), VA14_26 (purple), V1WT_41 (pink), and shared VA14_26/V1WT_41 contact residues (orange). The data underlying this Figure can be found in S1 Data.
(TIF)

S6 Fig Fab-RBD interaction depicted in context of specialist ACE2 utilising bat sarbecovirus Rc-o319. **A)** Superimposed 3D structure of WIV1 RBD-V1WT_06 Fab (blue) complex over Rc-o319 RBD (orange) - *Rh. cornutus* ACE2 (pink) complex (PDB: 9W77) [34]. **B)** Superimposed 3D structure of WIV1 RBD-VA14_26 Fab (red) complex over Rc-o319 RBD (orange) - *Rh. cornutus* ACE2 (pink) complex (PDB: 9W77) [34]. **C)** Superimposed 3D structure of WIV1 RBD-V1WT_41 Fab (green) complex over Rc-o319 RBD (orange) - *Rh. cornutus* ACE2 (pink) complex (PDB: 9W77) [34].
(TIF)

S7 Fig S1 shedding as a mechanism of SARS-CoV-2 neutralisation. Antibody mediated shedding of S1 from SARS-CoV-2 Spike. HEK 293T cells expressing Wuhan-1 Spike were incubated with mAbs and binding measured by flow cytometry at 5, 10, 20, 40, and 80 mins. mAb P008_67 was used as a positive control and VA14_47 was used as a negative control [65]. The data underlying this Figure can be found in S1 Data.
(TIF)

S8 Fig Binding of VA14_26 and V1WT_41 to WIV-1 RBD. **A)** Close up view of K378 on WIV-1 RBD and its contact residue on VA14_26. Panel on the right depicts the coulomb density mesh around the residues at SD level 9, as implemented in ChimeraX_1.7.1. **B)** Close up view of R408 on WIV-1 RBD and its contact residues on VA14_26. Panel on the right depicts the coulomb density mesh around the residues at SD level 10.25, as implemented in ChimeraX_1.7.1. **C)** Comparison in neutralisation potency of VA14_26 Fab and IgG against SARS-CoV-2 (D614G), SARS-CoV-1 and WIV-1. Mean values are plotted with error bars showing ± standard deviation (s.d.). **D)** Close up view of YYDRSG motif in V1WT_41 HCDR3 (green) in contact with WIV-1 RBD (grey), shown in cartoon representation. Regions outside the contact residues are turned transparent. Local structure with right-handed twist of beta hairpin is visible. **E)** Close up view of R408 on WIV-1 RBD and its contact residues on V1WT_41. Panel on the right depicts the coulomb density mesh around the residues at SD level 12.5, as implemented in ChimeraX_1.7.1. The data underlying this Figure can be found in S1 Data.
(TIF)

S1 Table Cryo-EM data collection, refinement, and validation statistics. (DOCX)

S1 Data All raw data that supports the figures. (XLSX)

S1 Raw image Raw image of western blot used in S1C Fig. Lysates of cells transfected with bat Sarbecovirus Spikes were boiled in nonreducing loading buffer for 5 min. SDS-PAGE and membrane-transfer was conducted as per standard methods. The membrane was blocked and incubated with primary mouse anti-FLAG tag antibody. For tagged Sarbecovirus Spikes, Spike expression was detected using secondary anti-mouse antibody, at expected band size of ~180 kDa. The blot was captured using LiCor Odyssey Fc using 700 nm (ladder) and 800 nm (sample) channel. The membrane was subsequently incubated with anti-GAPDH antibody and detected using a similar method at expected size of 36 kDa.
(TIF)

## Abbreviations

ACE2: angiotensin-converting enzyme 2
ADCC: antibody-dependent cellular cytotoxicity
bnAbs: broadly neutralising antibodies
BTI: breakthrough infection
CTF: contrast transfer function
DMEM: Dulbecco’s Modified Eagle Medium
FBS: foetal bovine serum
FSC: Fourier shell correlation
GMFI: geometric mean fluorescence intensity
HDX: hydrogen-deuterium exchange
hu-ACE2: human ACE2
IL: Interleukin
LuS: assembling lumazine synthase
mAbs: monoclonal antibodies
MEM-α: Minimum Essential Medium-α
MFI: mean fluorescence intensity
nAbs: neutralising antibodies
PID: percent identity
RBD: receptor binding domain
RBM: receptor binding motif
RU: response units
SPR: surface plasma resonance
