# Supplementary material for: Targeting of conserved spike epitopes enables broad antibody neutralisation of diverse bat sarbecoviruses
Source: PLoS Biol. 2026 Oct 1;24(10):e3003882. doi: 10.1371/journal.pbio.3003882 (PMC13630248; doi:10.1371/journal.pbio.3003882)
Supplement: Supplementary file 1 [file pbio.3003882.s009.docx]

**S1 Table:** Cryo-EM data collection, refinement and validation statistics

|  | **VA14_26–spike** complex | **V1WT_41**– **spike** complex | **V1WT_06**–**spike** complex |
| --- | --- | --- | --- |
| **Data collection and processing** |  |  |  |
| Microscope/ detector | Krios1/Falcon 4i | Krios1/Falcon 4i | Krios 2/Falcon 4i |
| Grid type | 300-mesh C-flat holey carbon grids (CF 1.2/1.3 ) | 300-mesh C-flat holey carbon grids (CF 1.2/1.3 ) | 300-mesh C-flat holey carbon grids (CF 1.2/1.3 ) |
| Voltage (kV) | 300 | 300 | 300 |
| Total exposure (e/Å) | 32.2 | 32.2 | 42 |
| Energy filter slit (eV) | - | - | 10 |
| Movies acquired | 4,500 | 4,500 | 7,058 |
| Defocus range (μm) | -1.5 to -3 | -1.5 to -3 | - 1.5 to -3.3 |
| Exposure time (s) | 5.44 | 5.44 | 5.44 |
| Pixel size (Å) | 1.08 | 1.08 | 0.95 |
| Number of EER frames | 1,674 | 1,674 | 1,674 |
| Calibrated Magnification | 75,000 | 75,000 | 130,000 |
| Dose rate ( e/Å^2^/s) | 5.92 | 5.92 | 7.53 |
| Total dose/Frame (e/Å^2^) | 32.2 | 32.2 | 41.0 |
| Particles picked  (Topaz autopick) | 1,460,644 | 1,446,370 | 3,514,633 |
| Particles used in final reconstruction | 86,961 | 139,733 | 101,825 |
| Map resolution (Å) | 4.12 | 3.57 | 3.98 |
| FSC threshold | 0.143 | 0.143 | 0.143 |
| **Refinement** |  |  |  |
| CCbox | 0.72 | 0.68 | 0.79 |
| CCmask | 0.74 | 0.78 | 0.74 |
| Clash score | 7.25 | 3.42 | 4.5 |
| Ramachandran favoured (%) | 94.74 | 95.46 | 96.33 |
| Ramachandran outliers (%) | 0 | 0 | 0 |
| Bond length (Å) | 0.003 | 0.003 | 0.002 |
| Bond Angle (°) | 0.665 | 0.571 | 0.542 |
| MolProbity clash score | 7.25 | 3.42 | 4.5 |
| MolProbity overall score | 1.76 | 1.76 | 1.82 |
